# Na^+^ entry through heteromeric TRPC4/C1 channels mediates (−)Englerin A-induced cytotoxicity in synovial sarcoma cells

**DOI:** 10.1038/s41598-017-17303-3

**Published:** 2017-12-05

**Authors:** Katsuhiko Muraki, Kaori Ohnishi, Akiho Takezawa, Hiroka Suzuki, Noriyuki Hatano, Yukiko Muraki, Nurasyikin Hamzah, Richard Foster, Herbert Waldmann, Peter Nussbaumer, Mathias Christmann, Robin S. Bon, David J. Beech

**Affiliations:** 1 Laboratory of Cellular Pharmacology, School of Pharmacy, Aichi-Gakuin University, 1-100 Kusumoto, Chikusa, Nagoya 464-8650 Japan; 2 School of Medicine, University of Leeds, Leeds, LS2 9JT UK; 3 School of Chemistry, University of Leeds, Leeds, LS2 9JT UK; 4 Institute of Chemistry and Biochemistry, Freie Universität Berlin, Takustraße 3, 14195 Berlin, Germany; 5 Max-Planck-Institut für Molekulare Physiologie, Otto-Hahn-Straße 11, 44227 Dortmund, Germany; 6 Technische Universität Dortmund, Fakultät für Chemie und Chemische Biologie, Otto-Hahn-Str. 6, D-44227 Dortmund, Germany; 7 Lead Discovery Center GmbH, Otto-Hahn-Str. 15, D-44227 Dortmund, Germany

## Abstract

The sesquiterpene (−)Englerin A (EA) is an organic compound from the plant *Phyllanthus engleri* which acts via heteromeric TRPC4/C1 channels to cause cytotoxicity in some types of cancer cell but not normal cells. Here we identified selective cytotoxicity of EA in human synovial sarcoma cells (SW982 cells) and investigated the mechanism. EA induced cation channel current (Icat) in SW982 cells with biophysical characteristics of heteromeric TRPC4/C1 channels. Inhibitors of homomeric TRPC4 channels were weak inhibitors of the Icat and EA-induced cytotoxicity whereas a potent inhibitor of TRPC4/C1 channels (Pico145) strongly inhibited Icat and cytotoxicity. Depletion of TRPC1 converted Icat into a current with biophysical and pharmacological properties of homomeric TRPC4 channels and depletion of TRPC1 or TRPC4 suppressed the cytotoxicity of EA. A Na^+^/K^+^-ATPase inhibitor (ouabain) potentiated EA-induced cytotoxicity and direct Na^+^ loading by gramicidin-A caused Pico145-resistant cytotoxicity in the absence of EA. We conclude that EA has a potent cytotoxic effect on human synovial sarcoma cells which is mediated by heteromeric TRPC4/C1 channels and Na^+^ loading.

## Introduction

Natural products have served as a source of chemical compounds for therapeutics. The sesquiterpene (−)Englerin A (EA) was discovered as a potent and selective inhibitor of renal cancer growth^1^ and it was later suggested that EA has anti-tumor activity via activation of a novel type protein kinase C (PKC), PKCθ^2^. In contrast, our previous studies have suggested that EA is a potent and selective activator of canonical transient receptor potential channel 4 (TRPC4) and 5 (TRPC5), and we have proposed that EA causes anti-tumor cell activity by Na^+^ loading into cells through heteromeric TRPC4/C1 channels^3,4^. Although Carson *et al*. have also shown that EA activates TRPC4 and TRPC5 to inhibit tumor cell growth^5^, a diterpene ester, tonantzitlolone can cause cytotoxicity in renal cancer cells via the activation of PKCθ^6^. Moreover, it is shown that EA modifies lipid metabolism and causes ER stress in renal cancer cells^7^. Therefore, it is widely discussed whether EA has multiple actions or a primary target after which follow multiple downstream mechanisms^8^. Although it has been recently shown that application of EA elevates cytosolic Ca^2+^ concentration in tumor and non-tumor cells, the large component of the elevation was relatively resistant to conventional inhibitors of TRPC4 and TRPC5, ML204 and clemizole (Clm)^9,10^.

The canonical transient receptor potential (TRPC) family, a sub family of the TRP superfamily, form cation channels as functional tetramers. Among the seven TRPCs, TRPC1, TRPC4, and TRPC5 are classified into the same TRPC subtype and they can compose either homotetrameric channels of TRPC4 or TRPC5 or heterotetrameric channels such as TRPC4/C1 and TRPC5/C1^11–13^. Since TRPC4 and TRPC5 are involved in diverse cellular functions (TRPC4: intestinal motility^14^, cardiac remodeling^15^, and visceral pain sensation^16^; TRPC5: neurite growth^17^, fear-related behavior^18^), they are potential targets of drugs to address dysfunction in these biological systems. However, the physiological functions of TRPC4 and TRPC5 are not fully understood at least in part because potent and selective pharmacological tools against TRPC1, TRPC4, and TRPC5 are limited, in particular blockers against heteromeric TRPC4/C1 and TRPC5/C1 channels have been lacking. However we recently reported that Pico145 is a powerful tool as a potent and selective inhibitor against heteromeric TRPC4/C1 and TRPC5/C1 as well as homometic TRPC4 and TRPC5^19^.

In the present study we report on cytotoxicity of EA to human synovial sarcoma SW982 cells. Synovial sarcoma, which accounts for 10–20% of soft-tissue sarcomas in adolescents and young adults, is a malignant neoplasm at almost any anatomic sites^20^. Although synovial sarcoma is moderately sensitive to cytotoxic chemotherapy with drugs like ifosfamide and anthracyclines^21,22^, drug resistance during the treatments has become more common. Hence, novel therapeutic strategies and new cytotoxic drugs are awaited. Based on the result of our study, we propose that heteromeric TRPC4/C1 is a primary target of EA for a potent anti-human synovial sarcoma cell effect mediated via Na^+^ loading caused by sustained channel activation coupled with insufficient compensation by Na^+^/K^+^-ATPase.

## Results

### Screening of EA-sensitive human cells

We first explored several human types of cell which express TRPC4 mRNA and respond to EA. We screened eight different types of tumor and non-tumor cells derived from human tissues by evaluating the TRPC4 mRNA gene expression with quantitative PCR (supplementary Fig. 1a) and measuring the functional expression of Ca^2+^-permeable TRPC4 and/or TRPC5 with Ca^2+^-response to 30 nM EA (supplementary Fig. 1b). Although among eight different types of cells employed, HaCaT, OUMS, HEK, and SW982 cells expressed TRPC4 mRNA transcripts more abundantly than IMR32, Caco2, A549, and PC3 cells, only SW982 cells significantly responded to 30 nM EA in Ca^2+^-measuring studies (0.57 ± 0.04 ΔF_340_/F_380_, six independent experiments, supplementary Fig. 1b and see an inset of supplementary Fig. 1b), suggesting that EA should target SW982 cell-viability.

### Concentration- and time-dependent EA-induced cell-death in SW982 cells

**Figure 1:**
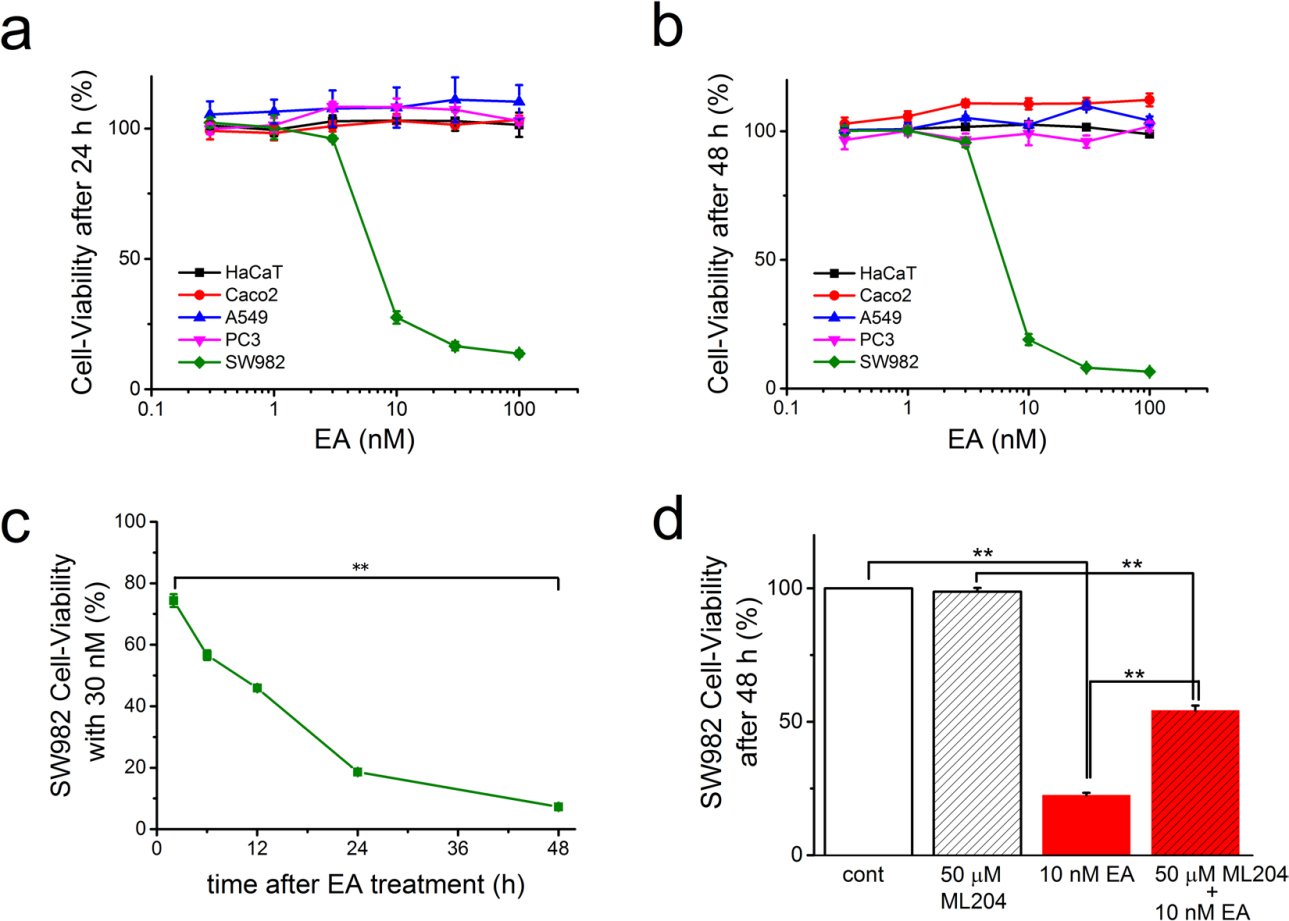
Concentration- and time-dependent EA-induced cell-death in SW982 cells. To examine whether EA causes cell-death in SW982 cells, EA at a concentration range between 0.3 nM and 100 nM was applied for 24 (**a**) and 48 h (**b**) and the cell-viability was assessed with WST-1 assay (three independent experiments). As comparisons, the same concentrations of EA were applied to HaCaT, Caco2, A549, and PC3 cells for 24 h and 48 h. (**c**) SW983 cells were exposed to 30 nM EA for 2, 6, 24, and 48 h and the cell-viability was assayed at each time (four independent experiments). (**d**) Effects of a putative TRPC4 inhibitor ML204 on EA-induced cell-death in SW982 cells. EA at 10 nM was applied for 48 h to SW982 cells pretreated with or without 50 μM ML204 (four independent experiments). Pooled data were averaged and expressed as mean ± SEM. The data were analyzed using one-way ANOVA (**c**) and Tukey’s test (**d**). **p < 0.01 compared with each group (**d**).

Since an exposure to EA effectively caused cell-death in a renal cancer cell line A498 (A498) and a breast cancer cell line Hs578T (Hs578T), both of which had an EA-induced activation of Icat^3,4^, we next examined whether EA treatments cause any cell-death in SW982 cells (Fig. 1). As shown in Fig. 1a and b, treatments with EA for 24 and 48 h markedly reduced the cell-viability in SW982 cells in a concentration-dependent manner, whereas the treatments had no effects on viability of HaCaT, Caco2, A549, and PC3 cells. Moreover, the exposure of SW982 cells to EA at 30 nM reduced the cell-viability in a time-dependent manner (Fig. 1c), demonstrating that EA potently and selectively causes cell-death in SW982 cells. On the other hand, pretreatment of SW982 cells with 50 μM ML204, a putative inhibitor of TRPC4 and TRPC5 (IC_50_ against TRPC4 and TRPC5 is ~1 μM and ~5 μM, respectively^23^), only in part attenuated the EA-induced cell-death (Fig. 1d), suggesting that ML204 is an effective but not efficient inhibitor for EA-induced cell-death in SW982 cells.

### Expression of TRPC subfamily TRPC1, TRPC4, and TRPC5 in SW982 cells

**Figure 2:**
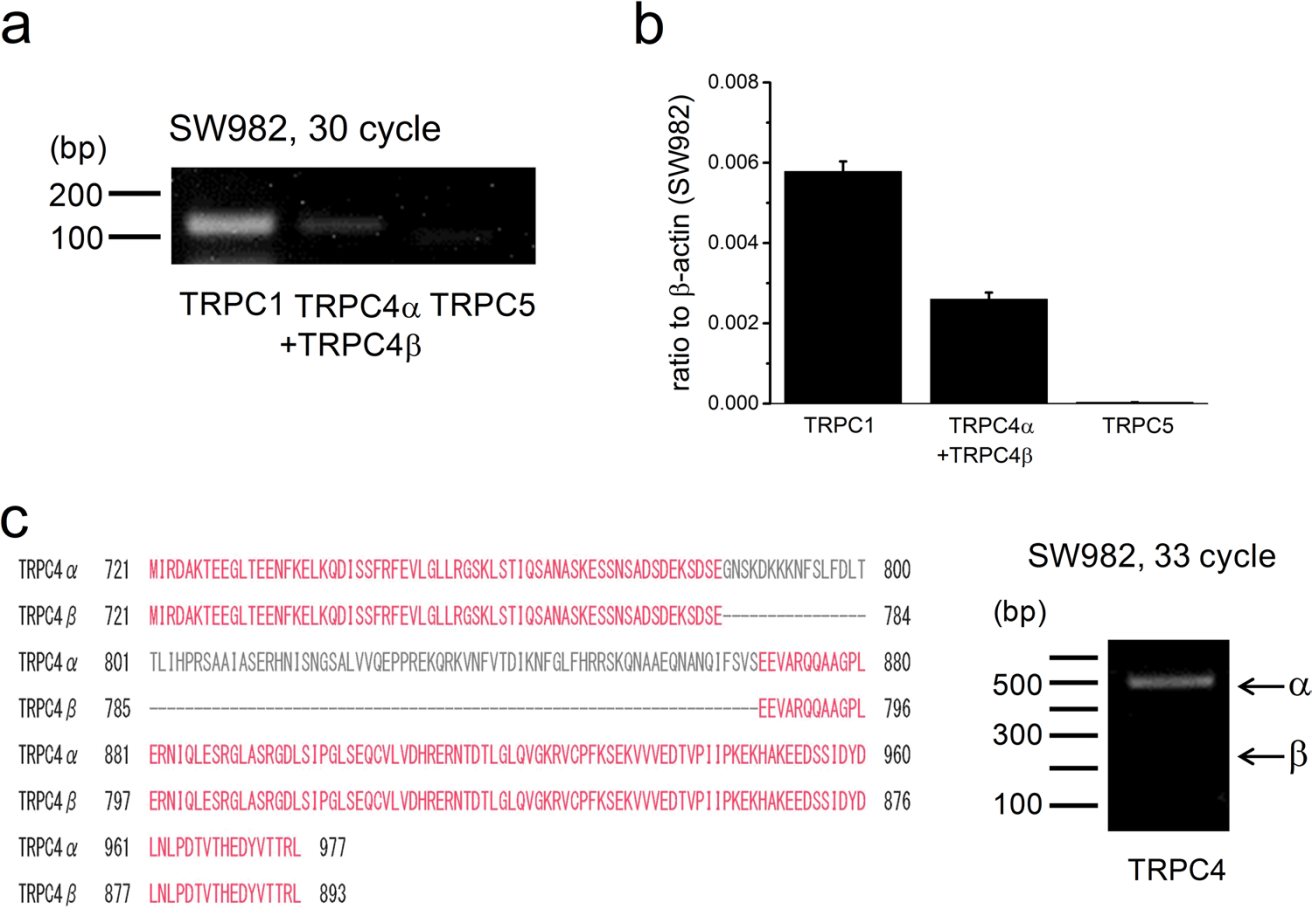
Expression of TRPC subfamily TRPC1, TRPC4, and TRPC5 in SW982 cells. The mRNA expression of TRPC1, TRPC4, and TRPC5 was determined in SW982 cells with RT-PCR (**a**) and quantitative PCR analysis (**b**). Pooled data were averaged and expressed as mean ± SEM (**b**). (**c**) The isoform expression level of TRPC4 (TRPC4α and TRPC4β) was also examined in SW982 cells with RT-PCR. Human TRPC4α protein has an insertion of 84 amino acids in the C-terminus of human TRPC4β protein. For the whole segment of gels ((**a**) and (**c**)) see the Supplementary Fig. 2.

An explanation for why ML204 is only a mild inhibitor of EA-induced cell-death is that heteromeric channel composition with TRPC1 and TRPC4 (TRPC4/C1) and/or TRPC1 and TRPC5 (TRPC5/C1) forms the EA-induced Icat in SW982 cells because TRPC1-containing channels are only weakly sensitive to ML204^19^. Therefore, we next examined expression of TRPC5 as well as TRPC1 and TRPC4 in SW982 cells. As shown in Fig. 2a and b, RT-PCR and quantitative PCR analysis revealed that TRPC1 and TRPC4 mRNA transcripts were clearly detected, while those of TRPC5 were not. Moreover, the expression level of TRPC4α isoform was more abundant than that of TRPC4β isoform in SW982 cells (Fig. 2c). These suggest that TRPC1 and TRPC4 are abundant in SW982 cells at mRNA levels.

### Biophysical properties of EA-induced Icat composed of homo- and heteromeric channel with TRPC1, TRPC4, and TRPC5

**Figure 3:**
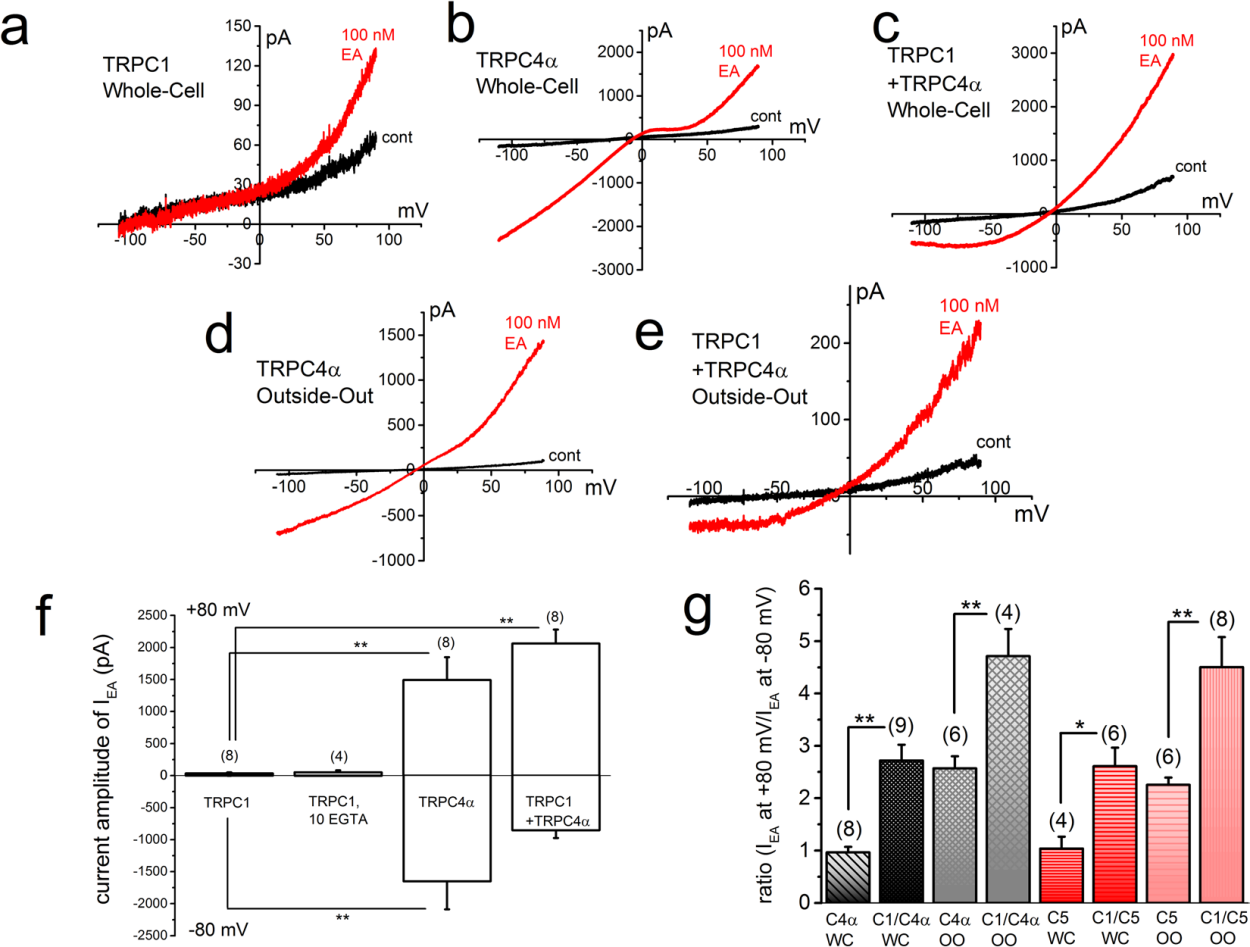
Biophysical properties of EA-induced Icat composed of homo- and heteromeric channel with TRPC1, TRPC4 and TRPC5. EA at 100 nM was applied to HEK cells expressing homomeric TRPC4α and TRPC5 and heteromeric TRPC4α/C1 and TRPC5/C1 under whole-cell and excised outside-out patch configurations. EA was also applied to HEK cells transfected with TRPC1. After whole-cell or outside-out patch recording was established, ramp waveform pulses from −110 to +90 mV for 400 ms were applied every 5 s. A typical I-V was exhibited in the presence and absence of EA in a HEK cell with TRPC1 (**a**), TRPC4α (**b**), and TRPC4α/C1 (**c**) under whole-cell conditions and in a HEK cell with TRPC4α (**d**) and TRPC4α/C1 (**e**) under outside-out patch conditions. (**f**) The peak amplitude of EA-induced Icat (I_EA_) at −80 and +80 mV was summarized in HEK cells with TRPC1, TRPC4α, and TRPC4α/C1 in whole-cell recordings. In HEK cells with TRPC1, 1 and 10 mM EGTA were dialyzed. The relative amplitude of I_EA_ at + 80 to I_EA_ at −80 mV was summarized as a ratio under each recording condition (**g**). Pooled data were averaged and expressed as mean ± SEM. The data were analyzed using Tukey’s test in (**f**) and student’s t-test in **(g)**. *p < 0.05 and **p < 0.01 compared with each group in (**f**) and with corresponding homomeric channel in (**g**). The number in parenthesis indicates the number of independent cells used.

In our previous study, EA potently activated both homomeric TRPC4β and heteromeric TRPC1 and TRPC4β^3^. However, it is not determined yet that EA can also activate channels with homomeric TRPC4α and heteromeric TRPC1 and TRPC4α (TRPC4α/C1). Therefore, we tested effects of EA on homomeric TRPC4α and heteromeric TRPC4α/C1, both of which were heterologously expressed in HEK cells. As comparisons, we also examined effects of EA on homomeric TRPC5 and heteromeric TRPC5/C1, both of which were used in our previous studies^3,19^. When 100 nM EA was applied to homomeric TRPC4α and heteromeric TRPC4α/C1 in whole-cell and excised outside-out patch configurations, the large amplitude of Icat was activated in which the current-voltage relationship (I-V) had a typical chair- and ladle-shape, respectively (Fig. 3b–f). In contrast, application of 100 nM EA to HEK cells expressing TRPC1 induced a tiny Icat which may be explained by endogenous channels (Fig. 3a and f). Vigorous internal dialysis with 10 mM EGTA, which may potentiate TRPC1-dependent currents, had no effect on EA-induced Icat in HEK cells with TRPC1 (Fig. 3f). Quantitative analysis of each current amplitude ratio between +80 and −80 mV revealed that both heteromeric TRPC4α/C1 and TRPC5/C1 have significantly larger rectification than homomeric TRPC4α and TRPC5 in whole-cell and excised outside-out patch configurations and these relatively large rectifications may be a potential characteristic of the heteromeric composition of these channels.

### Pharmacological properties of EA-induced Icat composed of homo- and heteromeric channel with TRPC1 and TRPC4

**Figure 4:**
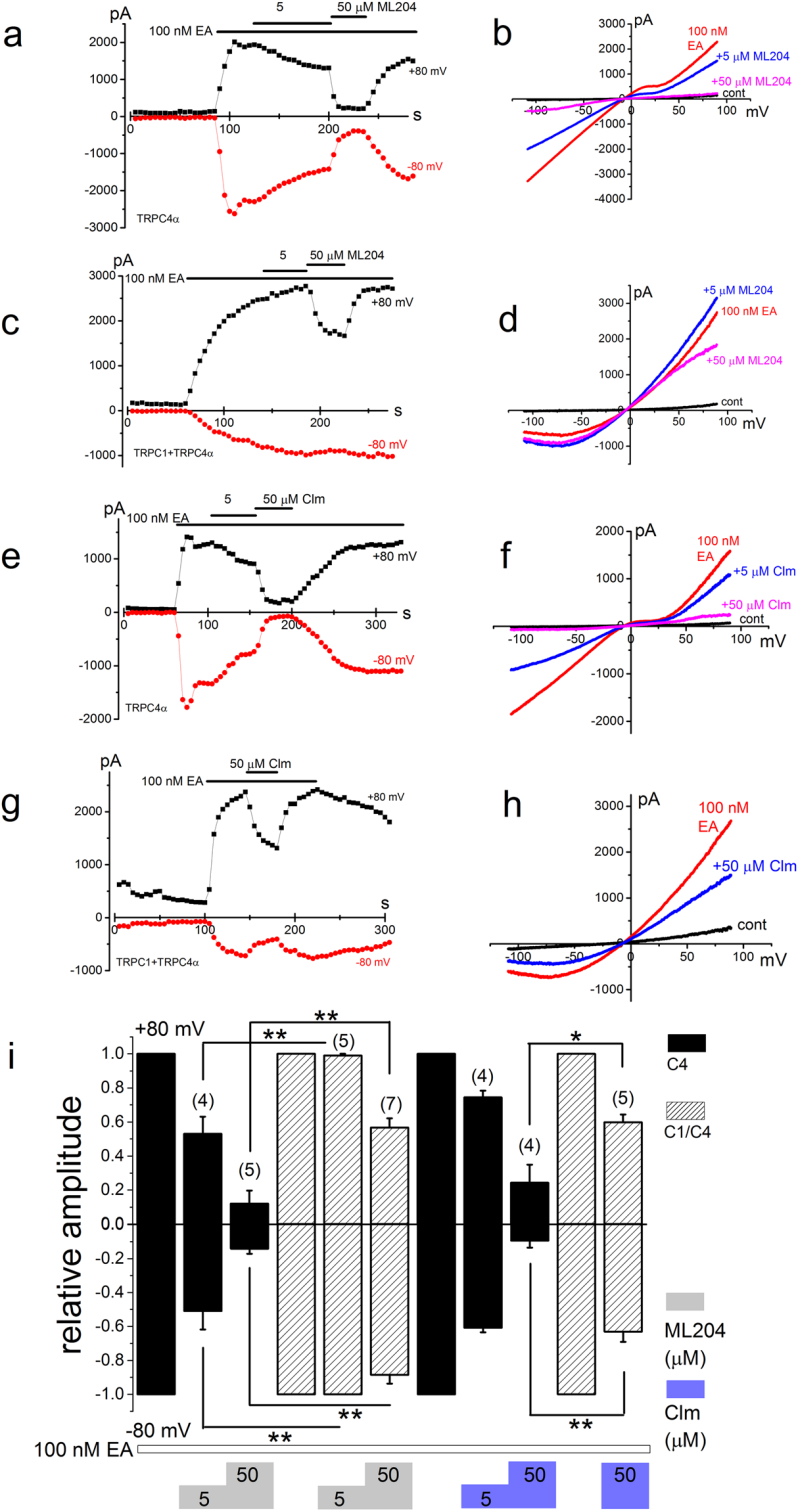
Pharmacological properties of EA-induced Icat composed of homo- and heteromeric channel with TRPC1 and TRPC4. After HEK cells with TRPC4α (**a,b** and **e,f**) and TRPC4α/C1 (**c,d** and **g,h**) were exposed to 100 nM EA, ML204 (**a,b** and **c,d**) and Clm (**e,f** and **g,h**) at 5 and 50 μM were applied. Ramp waveform pulses from −110 to + 90 mV for 400 ms were applied every 5 s and the peak amplitude of Icat at −80 and +80 mV was plotted against time (**a,c,e**, and **g**). A typical I-V was exhibited before and after application of 100 nM EA in the presence and absence of ML204 and Clm under whole-cell conditions (**b**,**d**,**f** and **h**). (**i**) The peak amplitude of I_EA_ at −80 and +80 mV in the presence of inhibitors was normalized to that in the absence under each recording condition. Pooled data were averaged and expressed as mean ± SEM. The data were analyzed using student’s t-test. *p < 0.05 and **p < 0.01 compared with homomeric TRPC4α. The number in parenthesis indicates the number of independent cells used.

Pharmacological profiles of homomeric TRPC4α and heteromeric TRPC4α/C1 were tested. Here we used two putative inhibitors against TRPC4, ML204 and Clm, whose IC_50_ values against TRPC4 were reported to be ~1 μM and ~6 μM, respectively^23,24^. Once the current amplitude at +80 mV had reached or was close to the steady-state in response to 100 nM EA, each cell was exposed to the inhibitors. As shown in Fig. 4a and b, 5 μM ML204 effectively inhibited homomeric TRPC4α, whereas it was much less effective against heteromeric TRPC4α/C1 (Fig. 4c and d). When 50 μM ML204 was applied, the inhibition of heteromeric TRPC4α/C1 was significant but less strong in comparison with homomeric TRPC4α (Fig. 4a,c and i). Another TRPC4 inhibitor Clm showed similar pharmacological profiles to ML204 (Fig. 4e–h): 50 μM Clm inhibited heteromeric TRPC4α/C1 less potently than homomeric TRPC4α (Fig. 4i). These results suggest that both ML204 and Clm are not potent inhibitors against heteromeric TRPC4α/C1, and in particular inward Icat composed of the heteromer seems resistant to even 50 μM ML204 and Clm.

### Biophysical and pharmacological properties of EA-induced Icat in SW982 cells

**Figure 5:**
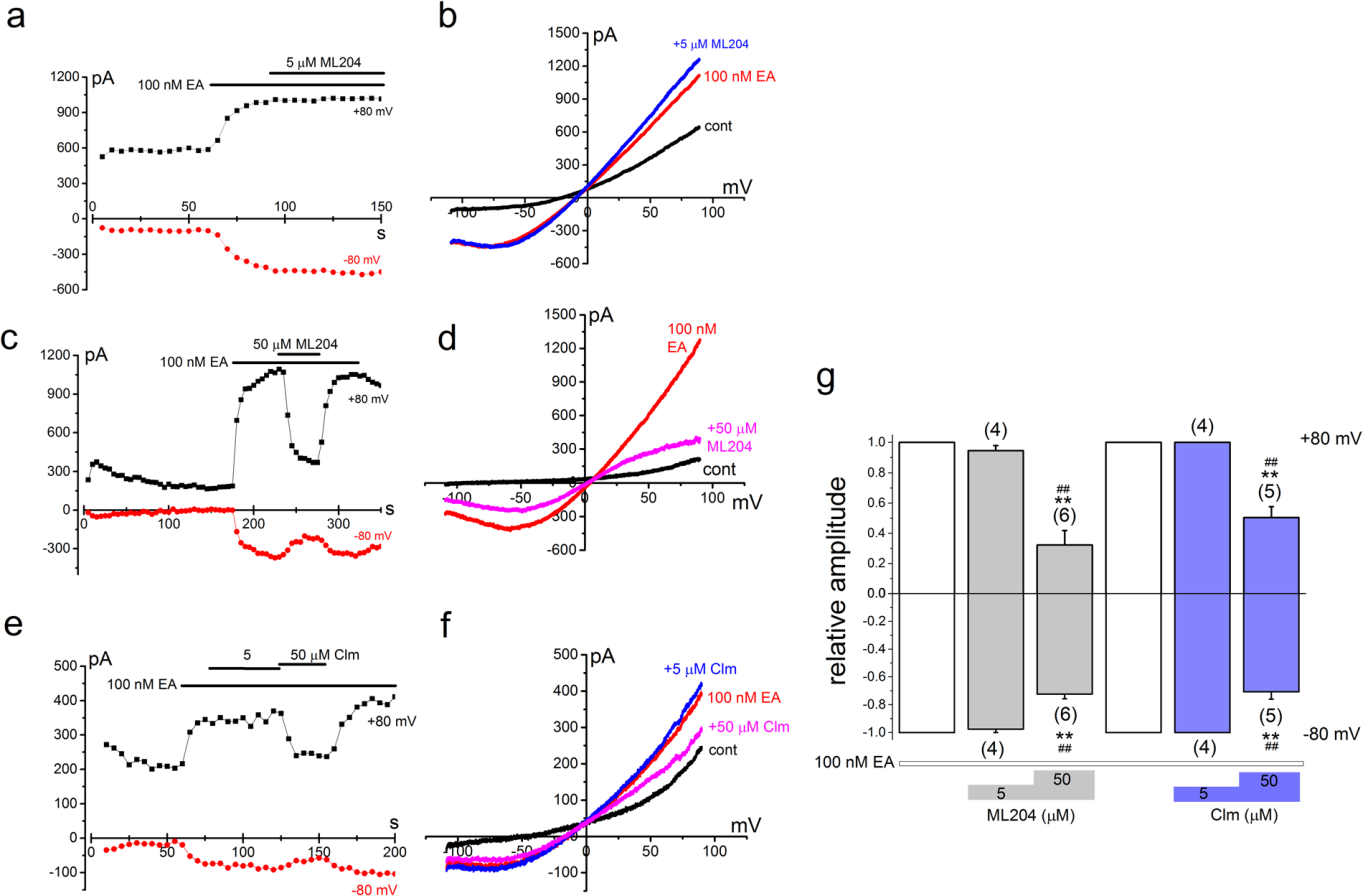
Biophysical and pharmacological properties of EA-induced Icat in SW982 cells. After SW982 cells were exposed to 100 nM EA, ML204 (**a–d**) and Clm (**e,f**) at 5 and 50 μM were applied. Ramp waveform pulses from −110 to + 90 mV for 400 ms were applied every 5 s and the change of peak amplitude of Icat at −80 and +80 mV was plotted against time (**a**, **c**, and **e**). A typical I-V was exhibited before and after application of 100 nM EA in the presence and absence of ML204 and Clm under whole-cell conditions (**b**, **d**, and **f**). (**g**) The peak amplitude of I_EA_ at −80 and +80 mV in the presence of inhibitors was normalized to that in the absence. Pooled data were averaged and expressed as mean ± SEM. The data were analyzed using Tukey’s test. **p < 0.01 and ^##^p < 0.01 compared with data without inhibitors and with 5 μM inhibitors, respectively. The number in parenthesis indicates the number of independent cells used.

**Figure 6:**
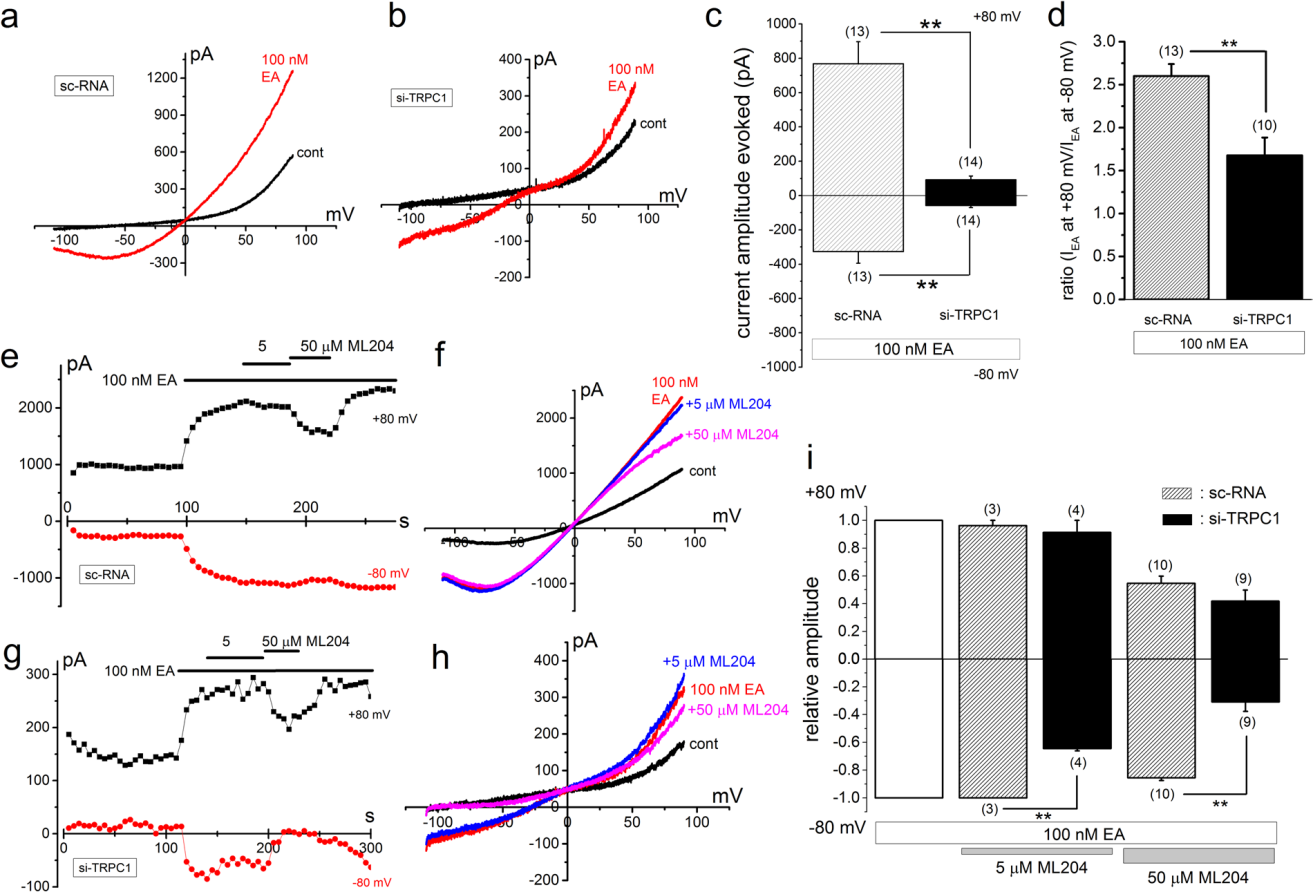
EA-induced Icat in SW982 cells depleted of TRPC1. EA at 100 nM was applied to SW982 cells pretreated with sc-RNA and si-TRPC1 to induce Icat. Ramp waveform pulses from −110 to + 90 mV for 400 ms were applied every 5 s. A typical I-V was exhibited in the presence and absence of EA in a SW982 cell pretreated with sc-RNA **(a)** and si-TRPC1 (**b**). The peak amplitude of EA-induced Icat (I_EA_) at −80 and +80 mV was summarized in SW982 cells pretreated with sc-RNA and si-TRPC1 (**c**). The relative amplitude of I_EA_ at +80 to I_EA_ at −80 mV was summarized as a ratio in SW982 cells pretreated with sc-RNA and si-TRPC (**d**). (**e**–**i**) After SW982 cells pretreated with sc-RNA and si-TRPC1 were exposed to EA, ML204 (5 and 50 μM) was applied and the change of peak amplitude of Icat at −80 and + 80 mV was plotted against time (**e** and **g**). A typical I-V was exhibited before and after application of EA in the presence and absence of ML204 in a SW982 cell pretreated with sc-RNA and si-TRPC (**f** and **h**). (**i**) The peak amplitude of I_EA_ at −80 and +80 mV in the presence of ML204 was normalized to that in the absence. Pooled data were averaged and expressed as mean ± SEM. The data were analyzed using student’s t-test. **p < 0.01 compared with SW982 cells with sc-RNA. The number in parenthesis indicates the number of independent cells used.

**Figure 7:**
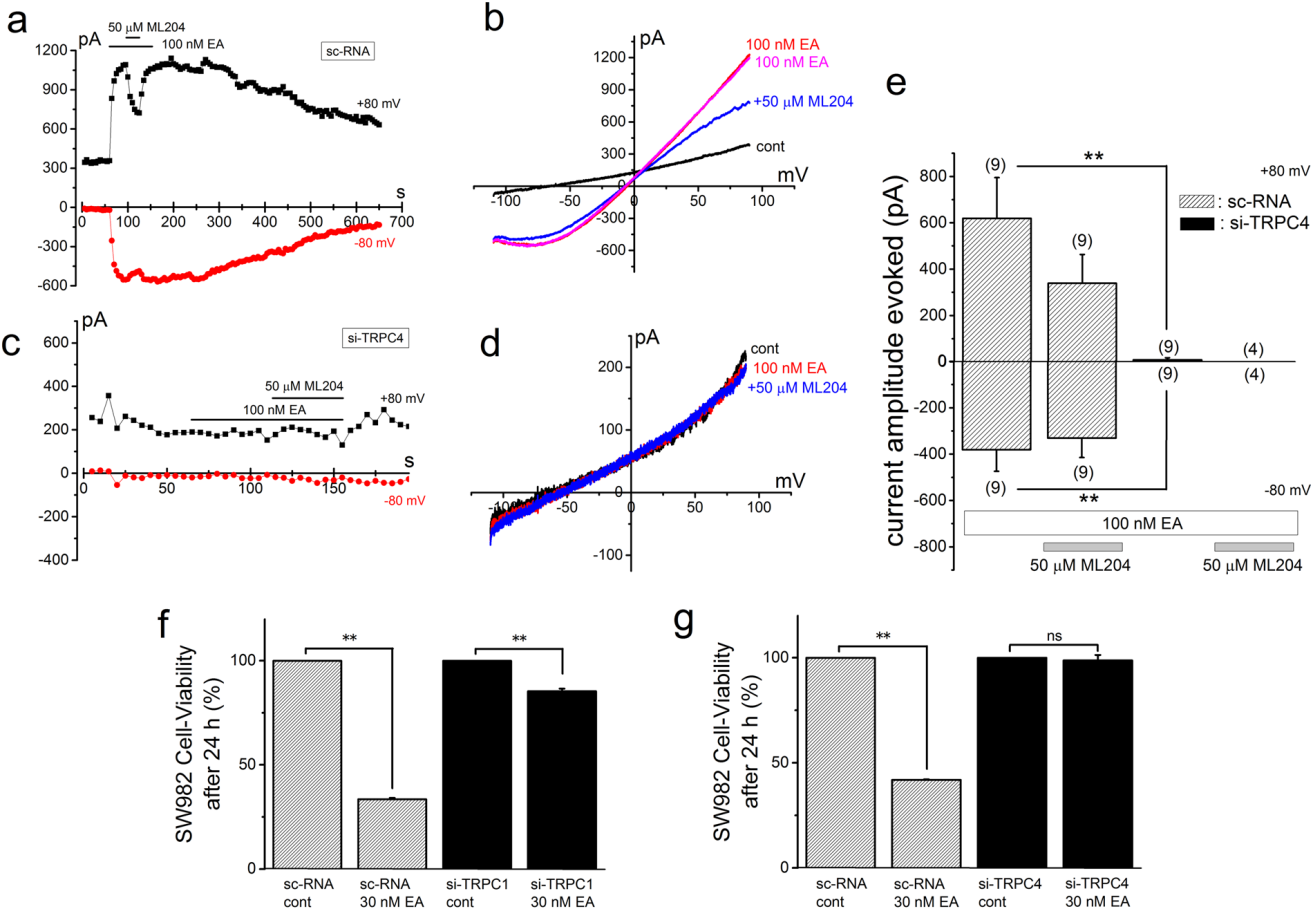
EA-induced Icat and cell-death in SW982 cells depleted of TRPC1 or TRPC4. (**a**–**e**) EA at 100 nM was applied to SW982 cells pretreated with sc-RNA and si-TRPC4 to induce Icat. Ramp waveform pulses from −110 to +90 mV for 400 ms were applied every 5 s and the change of peak amplitude of Icat at −80 and +80 mV was plotted against time (**a** and **c**). A typical I-V was exhibited in the presence and absence of EA in a SW982 cell pretreated with sc-RNA (**b**) and si-TRPC4 (**d**). The peak amplitude of EA-induced Icat (I_EA_) at −80 and +80 mV was summarized in SW982 cells pretreated with sc-RNA and si-TRPC4 (**e**). (**f,g**) Effects of EA on cell-death in SW982 cells pretreated with si-TRPC1 and si-TRPC4. EA at 30 nM was applied for 24 h to SW982 cells pretreated for 48 h with sc-RNA and si-TRPC1 (**f**) and with sc-RNA and si-TRPC4 (**g**) and the cell-viability was assayed (four independent experiments). Pooled data were averaged and expressed as mean ± SEM. The data were analyzed using student’s t-test. **p < 0.01 compared with SW982 cells with sc-RNA. The number in parenthesis indicates the number of independent cells used.

In Fig. 5, we applied EA to SW982 cells which were voltage-clamped under whole-cell conditions. EA at 100 nM activated Icat which had a clear ladle shape I-V (Fig. 5a and b). Moreover, the EA-induced current amplitude ratio between +80 and −80 mV was 2.13 ± 0.16 (n = 21) in SW982 cells, suggesting that the EA-induced Icat is composed of heteromeric TRPC4/C1. EA-induced Icat was resistant to both ML204 and Clm at 5 μM (Fig. 5a,e and g), while it was in part but significantly reduced by these inhibitors at 50 μM (Fig. 5c–g). These biophysical and pharmacological profiles of the EA-induced Icat suggest that EA treatment can activate heteromeric TRPC4/C1 in SW982 cells and that ML204 and Clm have limited usefulness as agents to test the importance of TRPC4/C1 in SW982 cell-viability. Therefore, we next used a gene knock-down technique to reduce TRPC1 and TRPC4 protein expressed in SW982 cells (Figs 6 and 7).

### EA-induced Icat and cell-death in SW982 cells depleted of TRPC1 and TRPC4

After SW982 cells were treated with sc-RNA and si-TRPC1 for 40–72 h, 100 nM EA was applied to activate Icat. Compared with a SW982 cell treated with sc-RNA (Fig. 6a), a cell with si-TRPC1 had a clear EA-induced chair-shape I-V, which is typical for homomeric TRPC4 (Fig. 6b). Moreover, the current amplitude of Icat in SW982 cells with si-TRPC1 was markedly reduced in comparison to that with sc-RNA (Fig. 6c) and the current ratio between −80 and +80 mV was significantly reduced to 1.68 ± 0.21 (n = 10) (Fig. 6d). Moreover, 5 μM ML204 effectively reduced Icat in SW982 cells with si-TRPC1 and the inhibition of Icat by 50 μM ML204 was significantly larger than that with sc-RNA (Fig. 6e–i). In contrast, when SW982 cells were treated with si-TRPC4, 100 nM EA failed to activate Icat (Fig. 7a–e), suggesting that TRPC1 does not form a functional channel in SW982 cells. We also examined effects of EA on cell-viability of SW982 cells in which either TRPC1 or TRPC4 was knocked-down by si-RNAs (Fig. 7f and g). When SW982 cells were treated with si-TRPC1, EA-induced cell-death was inhibited but still significant (Fig. 7f). In contrast, in SW982 cells treated with si-TRPC4, EA-induced cell-death was abolished (Fig. 7g). These data indicate that EA still activates a small size of Icat composed of homomeric TRPC4 in SW982 cells treated with si-TRPC1 (Fig. 6b) and hence EA can still cause death in these cells (Fig. 7f).

### Effects of Pico145 on EA-induced Icat and cell-death in SW982 cells

**Figure 8:**
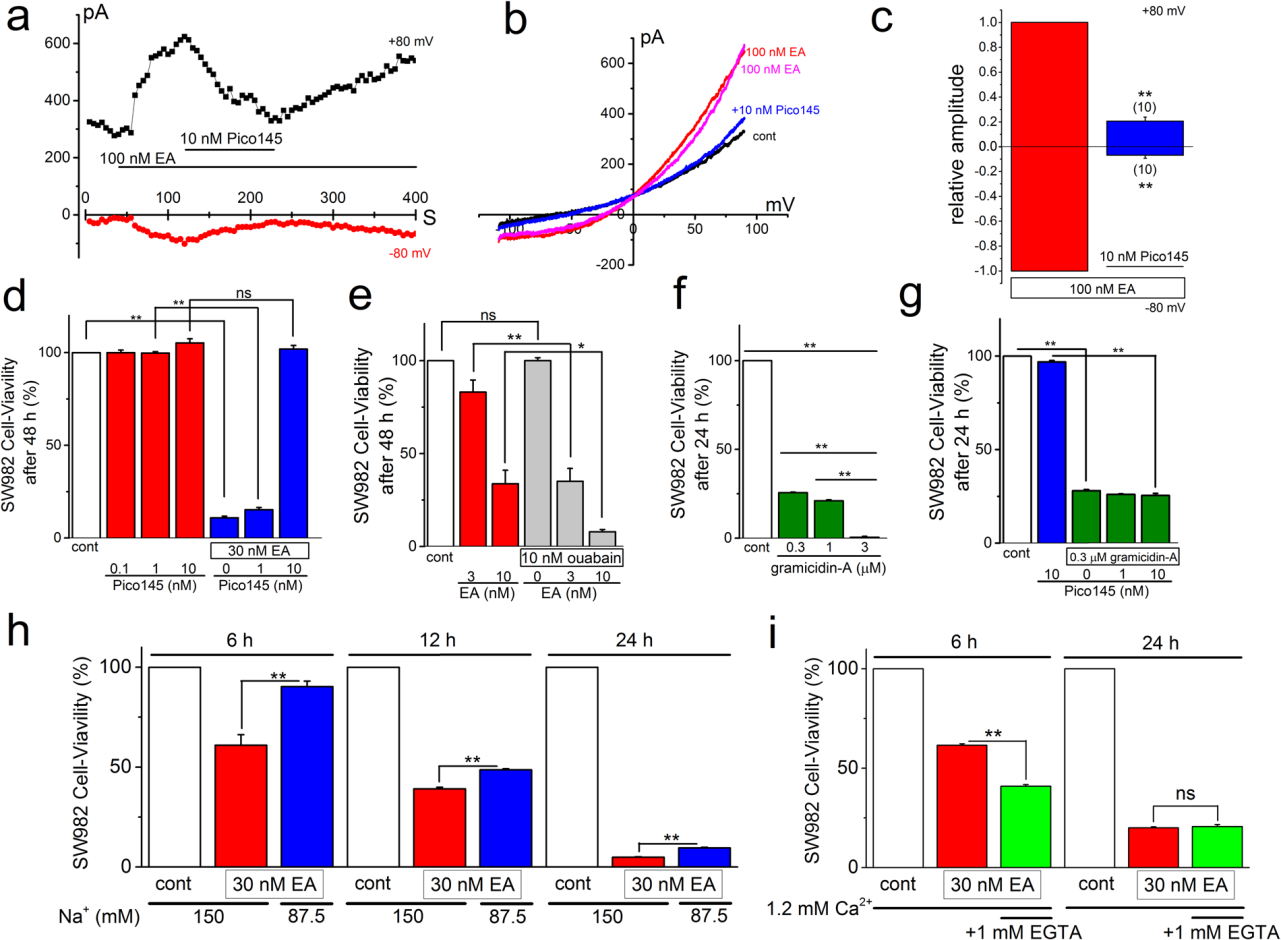
Effects of Pico145 on EA-induced Icat and cell-death in SW982 cells and Na^+^-dependent cytotoxicity. After SW982 cells were exposed to 100 nM EA, 10 nM Pico145 was applied. Ramp waveform pulses from −110 to + 90 mV for 400 ms were applied every 5 s and the change of peak Icat at −80 and +80 mV was plotted against time (**a**). A typical I-V was exhibited in the absence and presence of 100 nM EA before and during application, and after washout of Pico145 (**b**). (**c**) The peak I_EA_ at −80 and +80 mV in the presence of Pico145 was normalized to that in the absence and averaged. (**d**) Effects of Pico145 on EA-induced cytotoxicity of SW982 cells. EA at 30 nM was applied for 48 h with or without 1 and 10 nM Pico145 and the cell-viability was assayed (five independent experiments). (**e**) SW982 cells were exposed to 3 and 10 nM EA for 48 h with or without 10 nM ouabain and the cell-viability was assayed (four independent experiments). (**f**) SW982 cells were exposed to gramicidin-A for 24 h to test concentration-dependent effects on the cell-viability (four independent experiments). (**g**) SW982 cells were treated with 0.3 μM gramicidin-A for 24 h with or without 1 and 10 nM Pico145 (four independent experiments). (**h**,**i**) SW982 cells were exposed to 30 nM EA for 6, 12, and 24 h ((**h**), six (6 and 12 h) and four independent experiments (24 h)) in the Krebs solution with 150 and 87.5 mM Na^+^, and to 30 nM EA for 6 and 24 h ((**i**), six independent experiment each) in the Krebs solution with or without 1 mM EGTA, and each cell-viability was assayed. Na^+^ was replaced with the equimolar NMDG. Data were expressed as mean ± SEM and analyzed using student’s t-test (**c**–**e, g**–**i**), and Tukey’s test (**f**). *p < 0.05 and **p < 0.01 compared with SW982 cells without Pico145 (**c**), EA (**d**), ouabain (**e**), and gramicidin-A (**g**), and SW982 cells with lower external Na^+^ (**h**) and Ca^2+^ (**i**). The ‘ns’ shows no significance. The number in parenthesis indicates the number of independent cells used.

The gene-expression knock-down technique with si-RNAs is a powerful tool but may have a disadvantage of off-target effects by which expression of some proteins unrelated to the target gene are influenced^25^. To further confirm that the primary target of EA for cell-death in SW982 cells is heteromeric TRPC4/C1, our next maneuver involved Pico145, a novel potent inhibitor against heteromeric TRPC4/C1, which we have recently reported^19^. As shown in Fig. 8a–c, EA-induced Icat in SW982 cells was significantly inhibited in the presence of 10 nM Pico145, suggesting that Pico145 is a potent inhibitor against heteromeric TRPC4/C1 in SW982 cells. Pretreatment of SW982 cells with Pico145 between 0.1 and 10 nM in the absence of EA had little effect on the cell-viability (Fig. 8d). On the other hand, although the treatment of SW982 cells with 30 nM EA alone and together with 1 nM Pico145 caused substantial cell-death, the combination of 30 nM EA with 10 nM Pico145 failed to cause cell-death, strongly supporting the hypothesis that the primary target of EA in the cell-death is heteromeric TRPC4/C1.

### Na^+^-dependent cytotoxicity

We have previously shown that EA-evoked cytotoxicity against cancer cells is explainable by Na^+^ loading into cells via activation of TRPC4/C1^4^. Therefore, we next examined whether Na^+^ loading is involved in EA-evoked cytotoxicity of SW982 cells. As shown in Fig. 8e, EA-induced cell-death was significantly potentiated by the pretreatment of SW982 cells with a Na^+^-pump inhibitor ouabain (10 nM) as previously reported in other cells^4^. Moreover, a Na^+^ loading ionophore, gramicidin-A effectively caused the cell-death of SW982 cells in a concentration-dependent manner (Fig. 8f), which was resistant to 10 nM Pico145 treatments (Fig. 8g). Furthermore, when Na^+^ in the bathing solution was reduced to 87.5 mM by the replacement with the larger non-permeant cation N-methyl-D-glucamine (NMDG), EA-evoked cytotoxicity of SW982 cells was significantly inhibited: in particular, the cytotoxicity by 6 h EA-treatment was abolished (Fig. 8h). In contrast, the replacement itself (in the absence of EA) had little effect on each cell viability (94.9 ± 1.0%, 97.4 ± 1.0%, and 91.2 ± 1.5% cell viability of the control by the replacement for 6, 12, and 24 h, respectively). Since a large component of EA-evoked cytotoxicity for 24 h was relatively resistant to the reduction of Na^+^, we next tested the possible involvement of Ca^2+^ overload into cells by EA. However, addition of 1 mM EGTA to the bathing solution with 1.2 mM Ca^2+^ did not restore the EA-evoked cytotoxicity for 6 and 24 h (Fig. 8i). The addition of EGTA in the absence of EA had little effect on cell viability (94.2 ± 0.7% and 97.5 ± 1.4% cell viability of the control for 6 and 24 h). These results strongly suggest that Na^+^ loading is cytotoxic for SW982 cells.

## Discussion

In the present study we found that treatment of synovial sarcoma SW982 cells with EA effectively causes rapid and selective cytotoxicity in a concentration- and time-dependent manner. Biophysical, pharmacological, gene expression knocking-down and Na^+^ loading studies suggested that EA-induced cell-death is due to Na^+^ loading into cells via sustained activation of heteromeric TRPC4/C1 channels which is insufficiently compensated by Na^+^/K^+^-ATPase activity.

EA is a sesquiterpene extracted from *Phyllanthus engleri*. Although EA is a potent inhibitor of renal cancer cell growth, there are wide discussions regarding the mechanism involved^2–7,26–28^. In this study, we found that EA effectively caused cell-death in SW982 cells. In contrast, EA even at 100 nM had no effects on cell-viability of HaCat, Caco2, A549, and PC3 cells. Since 30 nM EA markedly reduces the cell-viability of SW982 cells to 10–20% for 24–48 h treatments, EA targets a soft-tissue sarcoma SW982 cell which expresses a potential functional molecule for EA. Similarly, EA caused cell-death of Ewing sarcoma cells, another soft-tissue sarcoma cell type^9^. On the other hand, although native HEK cells also expressed TRPC4 mRNA transcripts (supplementary Fig. 1a), these cells had no Ca^2+^ response to EA. It is possible that the full-length TRPC4 protein is not expressed in HEK cells. Consistently, control HEK cells were resistant to EA in our previous studies^3,4^.

A few molecular targets for potent EA cancer cell-death have been proposed: PKCθ^2^, TRPC4/C1 or TRPC4^3,5^, and sphingolipids^7^. High concentration of EA (>1 μM) blocks voltage-dependent L-type Ca^2+^ channels via an interaction with dihydropyridine binding sites^26^, but this effect is unlikely to be relevant to the cancer cell cytotoxic action of EA. EA-induced cancer cell-death was in part resistant to a TRPC4 inhibitor ML204 even at high concentrations (>50 μM^5,9^), suggesting that EA has multiple targets. Consistently, half of EA-induced cell-death in SW982 cells was resistant to 50 μM ML204 (Fig. 1d). Nevertheless, our present results support that EA mainly targets TRPC4/C1 in SW982 cells: it is found that ML204 is a poor inhibitor against heterometic TRPC4/C1 heterologously expressed in HEK cells and endogenously expressed in SW982 cells; a gene-knock down of TRPC4, an essential unit for channel function, abolished EA-induced Icat and cell-death in SW982 cells; pretreatment of SW982 with Pico145, a potent inhibitor of heteromeric TRPC4/C1, abolished EA-induced Icat and cell-death in SW982 cells. However, we cannot exclude the possibility of involvement of PKCθ and/or lipids in EA-induced cancer cell-death. Although the present study clearly showed the heteromeric TRPC4/C1 channel composition in SW982 cells, the stoichiometry is unknown. Since the mRNA expression of TRPC1 was more abundant than that of TRPC4 (Fig. 2b) and si-TRPC1 treatment reduced the EA-induced current amplitude of SW982 cells to 20% of the control (Fig. 6c), the involvement of one subunit of TRPC4 in the functional heterotetrameric TRPC4/C1 channel may be preferred by these cells.

We have recently proposed that Na^+^ loading into cells plays an important role in the underlying mechanism of EA-induced cancer cell-death^4^. Consistently, our present results support this mechanism: pretreatment of SW982 cells with a Na^+^ loading agent, ouabain substantially potentiated EA-induced cell-death. Moreover we provide the new information that application of the Na^+^ loading agent, gramicidin-A, caused cell-death in SW982 cells in a concentration-dependent manner. Gramicidin-A induced cell-death was totally resistant to Pico145 (Fig. 8g), importantly suggesting that Na^+^ loading itself is cytotoxic for cancer cells even without activation of TRPC4/C1. It is consistent that gramicidin-A treatment caused cell-death in several types of renal cancer cells^29^. On the other hand, the results were slightly complicated for experiments in which EA-induced cytotoxicity was tested in the bathing solution with lower Na^+^ concentration: the EA-induced cytotoxicity was abolished in 6 h treatment while in part sensitive to the reduction of Na^+^ in 12 and 24 h treatments. Since 87.5 mM Na^+^ was still present in the bathing solution under the present experimental conditions, the longer EA treatments may cause sufficient Na^+^ entry for cell-death. Alternatively, intracellular K^+^ loss as well as Ca^2+^ overload might be involved in EA-induced cytotoxicity. However, as previously described^4^, Ca^2+^ overload plays a minor role in EA-induced cytotoxicity of synovial sarcoma because addition of EGTA did not inhibit the EA-induced cell-death (Fig. 8i). Nevertheless, we cannot exclude the possibility that in later phase after exposure to EA, Na^+^-independent cytotoxicity might be unmasked. The mechanisms by which Na^+^ loading mediates cytotoxicity are not determined. Excess Na^+^ loading may deplete cellular ATP by forcing Na^+^/K^+^-ATPase into overdrive and inhibit a Na^+^-dependent amino acid transporter (system ASC transporter 2 (ASCT2)^30^) by reducing Na^+^ gradient. It is shown that ASCT2 activity is important for cancer cell proliferation^31^.

Synovial sarcoma accounts for 10–20% of soft-tissue sarcomas in adolescents and young adults^20^. Although synovial sarcoma is moderately sensitive to cytotoxic chemotherapy, drug resistance during the treatment has become more common and introduction of new drugs is clinically awaited. Since synovial sarcoma SW982 is highly sensitive to EA, EA may be promising as the basis for new agents for the treatment. Interestingly, among soft-tissue sarcomas, several Ewing sarcomas are also sensitive to EA^9^. For clinical application of EA and its analogues to these soft-tissue sarcomas, we should draw a final conclusion about EA-targets which have a strong connection to cancer pathology.

In conclusion, our results reveal that EA exhibits anti-tumor cell activity in SW982 cells and that heteromeric TRPC4/C1 channels in these cells are a critical target of EA. In addition, EA-induced cell-death in SW982 cells is mediated by Na^+^ loading via activation of heteromeric TRPC4/C1 channels coupled with insufficient Na^+^/K^+^-ATPase activity. Thus, EA may be a potential anti-tumor agent against soft-tissue synovial sarcoma and its effect may be potentiated by Na^+^/K^+^-ATPase inhibitors such as digoxin. Further study is needed to explore the therapeutic effects of EA and EA analogues on human samples of synovial sarcoma and tumors *in vivo*.

## Methods

### Reagents

The following drugs were used: (−)Englerin A (EA, AppliChem, Darmstadt, Germany), clemizole hydrochloride (Clm, BioVision, Milpitas, CA), 4-methyl-2-(1-piperidinyl)-quinoline (ML204, Tocris Bioscience, Bristol, United Kingdom), ouabain (Sigma/Aldrich, St. Louis, MO), Pico145^19^, and gramicidin-A (Sigma/Aldrich). Each drug except Pico145 was dissolved in the vehicle recommended by the manufacturer. Pico145 was dissolved in 100% DMSO to make 10 μM stoc^19^.

### Cell culture

Immortal human skin keratinocytes (HaCaT, American Type Culture Collection: ATCC, Manassas, VA), human chondrosarcoma (OUMUS, Health Science Research Resources Bank: HSRRB, Osaka, Japan), human colorectal adenocarcinoma (Caco2, DS Pharma Biomedical Co., Osaka, Japan), human alveolar A549 (A549, DS Pharma Biomedical Co., Osaka Japan), human prostate adenocarcinoma (PC3, kindly gifted by Professor Ohya), human synovial sarcoma SW982 (SW982, ATCC), human embryonic kidney 293 cell-line (HEK, HSRRB), and human neuroblastoma IMR32 (IMR32, DS Pharma Biomedical Co.) were maintained in culture medium recommended by the manufacturer. All culture medium was supplemented with 10% heat-inactivated FCS (GIBCO, Waltham, MA), streptomycin (100 μg/mL, Meiji Seika Pharma Co., Ltd., Tokyo, Japan), and penicillin G (100 U/mL, Meiji Seika Pharma Co., Ltd.).

### Recombinant expression of TRPCs in HEK cells

Partially confluent HEK cells (40–60% confluency) were transfected with the pcDNA3.1 (TRPC4α for screening in supplementary Fig. 1), pIRES2-AcGFP1 (TRPC1, TRPC4α, and TRPC5) and pIRES2-DsRed-Express2 (TRPC4α and TRPC5 for heteromeric channel expression) plasmids containing human TRPC1, TRPC4α, and TRPC5 cloned by ourselves, respectively, using lipofectamine 3000 (ThermoFisher Scientific, Yokohama, Japan). All constructs were sequenced. All experiments were performed within 48 h after transfection.

### Reverse transcription-PCR

RT-PCR amplification for TRPC1, TRPC4, TRPC4α, TRPC4β, and TRPC5 was performed as described previously^32^. The thermal cycler program used for PCR amplification included a 30 s denaturation step at 94 °C, a 30 s annealing step at 55 °C, and a 30 s primer extension step at 72 °C for 30 and 33 cycles using an ABI 2720 thermal cycler (Applied Biosystems, Foster City, CA). The amplified products were separated on 1.5% agarose gels in Tris acetate/EDTA buffer, visualized with 1 μg/ml ethidium bromide, and assessed on FAS III (TOYOBO, Osaka, Japan). Oligonucleotide sequences of primers specific for human TRPC1, TRPC4, TRPC4α, TRPC4β, and TRPC5 are shown in supplementary Table 1.

### Quantitative PCR

Real-time quantitative PCR was done with the use of SYBR Green Chemistry on a Thermal Cycler Dice Real Time System (Takara Bio, Inc., Kusatsu, Japan) as described previously^32^. Transcriptional quantification of gene products was normalized to that of β-actin and each cDNA sample was tested in triplicate. The program used for quantitative PCR amplification included a 30 s activation of Ex Taq™ DNA polymerase at 95 °C, a 15 s denaturation step at 95 °C, a 60 s annealing and extention step at 60 °C (for 45 cycles), and a dissociation step (15 s at 95 °C, 30 s at 60 °C and 15 s at 95 °C). Oligonucleotide sequences of primers specific for human TRPC1, TRPC4, TRPC5, and β-actin are shown in supplementary Table 1.

### Patch clamp experiments

Whole-cell and excised single channel recording experiments were performed as described previously^33^. The resistance of pipettes was 3–5 MΩ when filled with pipette solution. A Cs^+^ rich pipette solution contained [in mM] Cs-aspartate 110, CsCl 30, MgCl_2_ 1, HEPES 10, EGTA 1 or 10, and Na_2_ATP 2 [adjusted to pH 7.2 with CsOH]. Membrane currents and voltage signals were digitized using an analogue-digital converter (PCI6229, National Instruments Japan, Tokyo, Japan) driven by WinWCPV4.5 for data acquisition and analysis of whole-cell currents and excised outside-out single channel currents (developed by Dr. John Dempster, University of Strathclyde, UK). The liquid junction potential between the pipette and bath solutions (−10 mV) was corrected. A ramp voltage protocol from −110 mV to + 90 mV for 400 ms was applied every 5 s from a holding potential of −10 mV. A leak current component was not subtracted from the recorded currents. A standard HEPES-buffered bathing solution (SBS [in mM]: NaCl 137, KCl 5.9, CaCl_2_ 2.2, MgCl_2_ 1.2, glucose 14, HEPES 10 [adjusted to pH 7.4 with NaOH]) was used. All experiments were performed at 25 ± 1 °C.

### Measurement of Ca^2+^ fluorescence ratio

Cells, which were loaded with 10 μM Fura2-AM (Dojindo, Kumamoto, Japan) in the SBS for 30 min at room temperature, were superfused with SBS for 10 min and then Fura-2 fluorescence signals were measured at 0.1 Hz using the Argus/HisCa imaging system (Hamamatsu Photonics, Hamamatsu, Japan) driven by Imagework Bench 6.0 (INDEC Medical Systems, Santa Clara, CA). In each analysis, a whole cell area was chosen as a region of interest to average the fluorescence ratio.

### Specific knockdown of TRPC1 and TRPC4 by RNA Interference

The sequence of the stealth short interference RNA (siRNAs) duplex oligoribonucleotides against human TRPC1 (si-TRPC1, GenBank Accession No. NM_001251845.1, Invitrogen, Carlsbad, CA) and TRPC4 (si-TRPC4, GenBank Accession No. NM_003306.1 Invitrogen) is 5′-CCUUUGCCCUCAAAGUGGUUGCUCA-3′ for sense strand and 5′-UGAGCAACCACUUUGAGGGCAAAGG-3′ for antisense strand, and 5′-GGAAGAACUGCUCUCCUCAUUGCAA-3′ for sense strand, and 5′-UUGCAAUGAGGAGAGCAGUUCUUCC-3′ for antisense strand, respectively. As a negative control for the siRNAs treatment, Medium GC Stealth RNAi Negative Control Duplex (sc-RNA, Invitrogen) was used. The cells in a 35-mm dish and in 24-well plate were washed with fresh culture medium without antibiotics 3 h prior to transfection. The si-RNAs or sc-RNA (20 µM, 6 µl for 35-mm dish and 1.5 μl for 24-well plate) and Lipofectamine RNAiMAX (5 µl for 35-mm dish and 1.25 μl for 24-well plate, Invitrogen) were diluted in 200 µl (35-mm dish) and 50 μL (24-well plates) Opti-MEM (Invitrogen), respectively. The diluted si-RNAs or sc-RNA solution and the diluted Lipofectamine RNAiMAX solution were mixed. These two mixtures were combined and incubated for 20 min at room temperature for complex formation. The entire mixture was added to the cells, resulting in a final concentration of 50 nM for both si-RNAs and sc-RNA. The cells were incubated for 40–72 h in a CO_2_ chamber.

### WST-1 cell-viability assay

Cells were seeded onto 24-well plates 24 h prior to WST-1 measurements (1 × 10^4^ SW982, 1 × 10^4^ HaCaT, 4 × 10^4^ Caco2, 5 × 10^3^ A549, and 1 × 10^4^ PC3 cells). The cell proliferation reagent WST-1 (Roche Applied Science, Penzberg, Germany) was used in accordance to the manufacturer’s instructions. Reduction of the tetrazolium salt WST-1 to formazan by mitochondrial dehydrogenases was determined by absorbance measurement at 450 nm (DXT880 Multimode Detector, Beckman Coulter, Brea, CA). Cell-death, loss of mitochondrial dehydrogenase activity, was inferred from a decrease in this reaction. Background absorbance at the reference wavelength 620 nm was subtracted. Each cell-sample was tested in duplicate or triplicate and pooled data were summarized in independent experiments. When the cell-viability was assayed in lower Na^+^ and Ca^2+^ bathing solution (Fig. 8h and i), Krebs buffer solution was used (Krebs [in mM]: NaCl 125, KCl 3.8, CaCl_2_ 1.2, MgSO_4_ 1.5, glucose 8, KH_2_PO_4_ 1.2, and NaHCO_3_ 25 (pH 7.4)).

### Statistical analyses

Data are expressed as the mean ± SEM except supplementary Fig. 1b (mean ± SD). Statistical significance between two groups and among multiple groups was examined using paired or unpaired Student’s t-tests, and ANOVA or Tukey’s test, respectively, which were two-tailed (Origin J9.1, LightStone, Tokyo, Japan). For all tests, P values less than 0.05 were considered statistically significant.

## Electronic supplementary material

Supplementary Info
