# Supplementary material for: Na+ entry through heteromeric TRPC4/C1 channels mediates (−)Englerin A-induced cytotoxicity in synovial sarcoma cells
Source: Sci Rep. 2017 Dec 5;7:16988. doi: 10.1038/s41598-017-17303-3 (PMC5717101; doi:10.1038/s41598-017-17303-3)
Supplement: Supplementary file 1 — Supplementary Info [file 41598_2017_17303_MOESM1_ESM.pdf]

Supplementary information

Na^+ entry through heteromeric TRPC4/C1 channels mediates (-)Englerin A-induced cytotoxicity in synovial sarcoma cells

Katsuhiko Muraki[1*], Kaori Ohnishi[1#], Akiho Takezawa[1#], Hiroka Suzuki[1#], Noriyuki Hatano[1], Yukiko Muraki[1], Nurasyikin Hamzah[3], Richard Foster[3], Herbert Waldmann[5,6], Peter Nussbaumer[7], Mathias Christmann[4], Robin S Bon[2], David J Beech[2]

[1]Laboratory of Cellular Pharmacology, School of Pharmacy, Aichi-Gakuin University, 1-100 Kusumoto, Chikusa, Nagoya 464-8650, Japan, [2]Schools of Medicine and [3]Chemistry, University of Leeds, Leeds, LS2 9JT, UK, [4]Institute of Chemistry and Biochemistry, Freie Universität Berlin, Takustraße 3, 14195 Berlin, Germany, [5]Max-Planck-Institut für Molekulare Physiologie, Otto-Hahn-Straße 11, 44227 Dortmund, Germany, [6]Technische Universität Dortmund, Fakultät für Chemie und Chemische Biologie, Otto-Hahn-Str. 6, D-44227 Dortmund, Germany [7]Lead Discovery Center GmbH, Otto-Hahn-Str. 15, D-44227 Dortmund

# These authors equally contributed to this work.

*Correspondence and requests for materials should be addressed to Katsuhiko Muraki (kmuraki@dpc.agu.ac.jp, Tel: +81-52-757-6788, Fax: +81-52-757-6799)

Supplementary Table1.

Each primer sequence used in this study is summarized.

target	Accession No.	sequence (F: forward, R: reverse)	product size (bp)
human TRPC1	NM_001251845.1	F: CACCTGTCATTTTAGCTGCTCATC	143
		R: CCGGAGGCTATCCTTTTTGTT	
human TRPC4	NM_016179.2	F: AGGTACTCTGCCTACTCCCTTCAA	141
		R: GCAGCTCGCCTCCCTATTG	
human TRPC4	NM_016179.2<br>NM_001135955.1	F: TCTTCAAATTCGGCAGACTCA	506 (TRPC4α)<br>254 (TRPC4β)
		R: TCCTCCACCACCACCTTCTC	
human TRPC5	NM_012471.2	F: TGAGTTCAAGGCCGAGTATGAG	114
		R: TCTCGATGGTTGAGGATGATCTC	
human β-actin	NM_001101.3	F: ACCGAGCGCGGCTACA	112
		R: CAGCCGTGGCCATCTCTT	

supplementary Table 1

Supplementary Figure1. Screening of EA-sensitive human cells.

To screen EA-sensitive human cells, eight different types of tumor and non-tumor cells were employed. (**a**) Evaluation of TRPC4 mRNA gene expression with quantitative PCR. Each expression was normalized to that of β-actin and summarized as mean ± SE (three independent experiments). (**b**) To measure the functional expression of Ca^{2+}-permeable TRPC4 and/or TRPC5, 30 nM EA was applied to eight human types of cells with measuring Ca^{2+}-response. The peak change in Ca^{2+} response of all individual cells was plotted as a scattered graph in six independent experiments each. When cells had larger EA-response than a threshold (0.05 ratio), the pooled data were averaged and expressed as mean ± SD. The total (N) and responded (n) number of cells were shown as n/N in the figure and a typical change in Ca^{2+} response of a SW982 cell was imposed in the inset. The data were analyzed using student's t-test. **p<0.01 compared with the threshold (0.05).

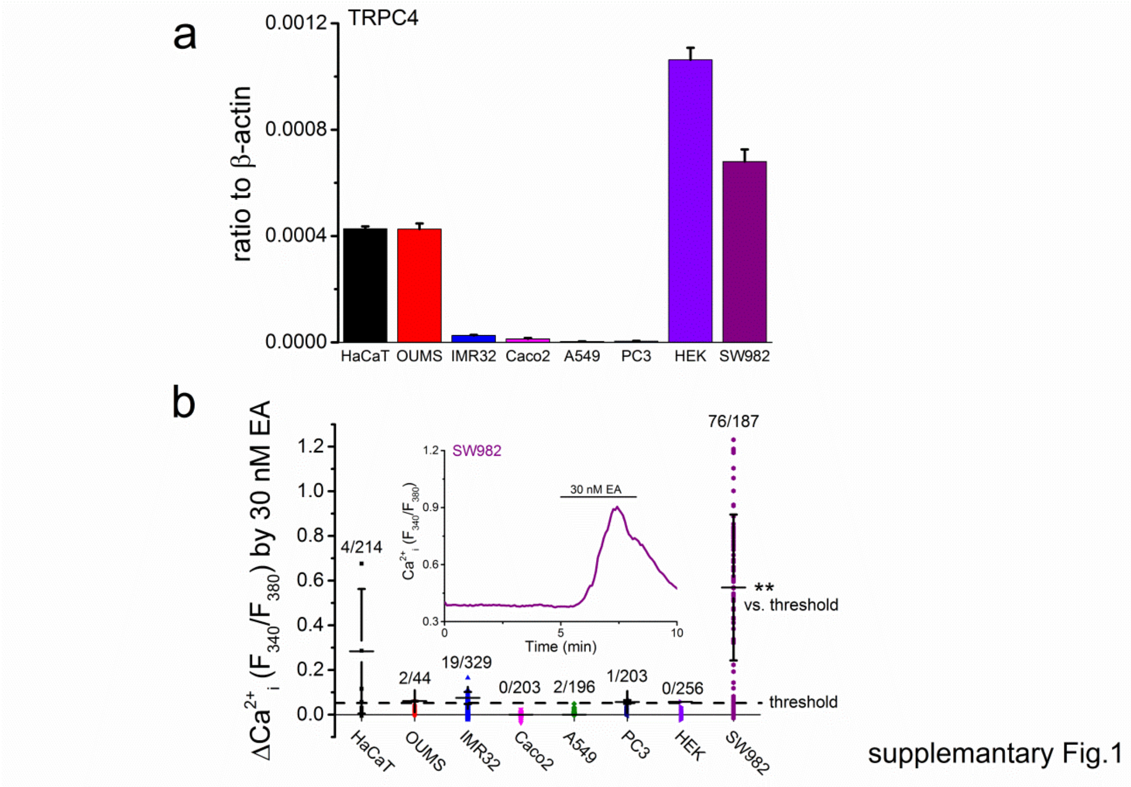

Supplementary Figure2.

The whole segment of gels in Fig.2 was shown. (**a**) The mRNA expression of TRPC1, TRPC4, and TRPC5 was determined in SW982 cells with RT-PCR. (**b**) The isoform expression level of TRPC4 (TRPC4α and TRPC4β) was also examined in SW982 cells with RT-PCR.

a

SW982, 30 cycle

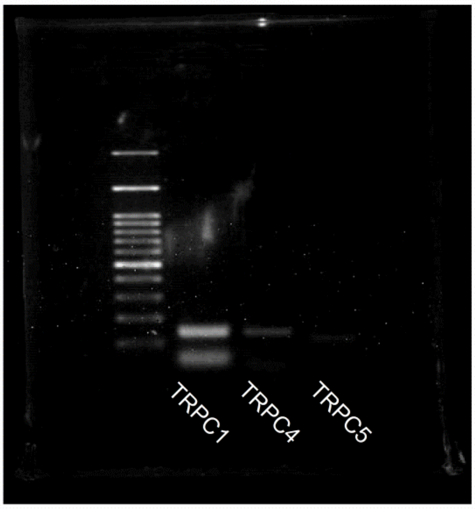

b

SW982, 33 cycle

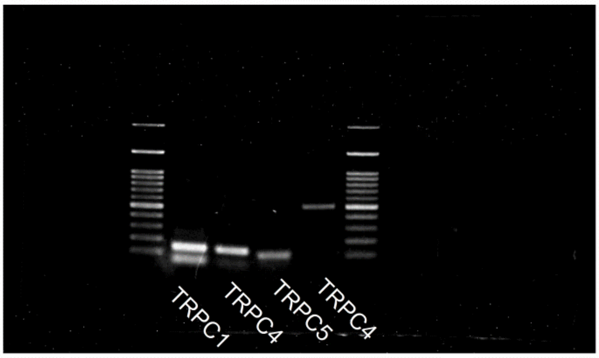

Supplementary Fig.2
